# Mesenchymal stem cell-derived extracellular vesicles ameliorate Alzheimer's disease-like phenotypes in a preclinical mouse model

**DOI:** 10.7150/thno.62069

**Published:** 2021-07-13

**Authors:** Allaura S. Cone, Xuegang Yuan, Li Sun, Leanne C. Duke, Michael P. Vreones, Allison N. Carrier, Stephanie M. Kenyon, Spencer R. Carver, Sarah D. Benthem, Alina C. Stimmell, Shawn C. Moseley, David Hike, Samuel C. Grant, Aaron A. Wilber, James M. Olcese, David G. Meckes

**Affiliations:** 1 Department of Biomedical Sciences, Florida State University College of Medicine, Tallahassee, FL 32306, USA; 2 Department of Chemical and Biomedical Engineering, Florida A&M University and Florida State University College of Engineering, Tallahassee, FL 32306, USA; 3 The National High Magnetic Field Laboratory, Florida State University, Tallahassee, Florida 32310, USA; 4 Department of Neuroscience, Florida State University College of Psychology, Tallahassee, FL 32306, USA

**Keywords:** Alzheimer's disease, extracellular vesicles, exosomes, microvesicles, mesenchymal stem cells

## Abstract

Alzheimer's disease (AD) is an irreversible neurodegenerative disorder that affects more than 44 million people worldwide. Despite the high disease burden, there is no effective treatment for people suffering from AD. Mesenchymal stem cells (MSCs) are multipotent stromal cells that have been widely studied due to their therapeutic potential. However, administration of cells has been found to have a multitude of limitations. Recently, extracellular vesicles (EVs) derived from MSCs have been studied as a therapeutic candidate, as they exhibit similar immunoprotective and immunomodulatory abilities as the host human MSCs.

**Methods:** To test the potential therapeutic effects of MSC EVs, human bone-marrow derived MSCs were grown in three-dimensional (3D) cell culture, and small EVs were harvested using differential ultracentrifugation. These small EVs were given to non-transgenic (NT) or 5XFAD (5 familial Alzheimer's disease mutations) mice intranasally (IN) every 4 days for 4 months. The mice were then required to perform a variety of behavioral assays to measure changes in learning and memory. Afterwards, immunohistochemistry was performed on brain slices to measure amyloid beta (Aβ) and glial fibrillary acidic protein (GFAP) levels.

**Results:** The data revealed that 5XFAD mice that received hMSC-EV treatment behaved significantly better in cognitive tests than saline treated 5XFAD mice, with no significant change between EV-treated 5XFAD mice and NT mice. Additionally, we found lower Aβ plaque load in the hippocampus of the EV-treated mice. Finally, less colocalization between GFAP and Aβ plaques was found in the brain of EV-treated mice compared to saline.

**Conclusions:** Taken together, these data suggest that IN administration of MSC-derived EVs can slow down AD pathogenesis.

## Introduction

Alzheimer's disease (AD) is the most common neurodegenerative disorder. It is characterized by loss of memory and behavioral changes, along with the formation of senile plaques and neurofibrillary tangles in the patient's brain 1. It is estimated that 5.8 million Americans live with AD in the United States, and the number is expected to rise to 14 million by 2050 2-4. It is estimated that 14% of people 71 and older are living with AD 2. Despite the high disease burden, the exact cause of AD is still unknown. There is no known cure or even an effective treatment for people suffering from this disease.

One of the possible causes of AD is proposed by the amyloid beta (Aβ) hypothesis, where Aβ oligomerizes to form insoluble plaques that lead to neuronal cell death. Aβ forms when amyloid precursor protein (APP) is cleaved by a β-secretase to form the C99 fragment and soluble APP β (sAPPβ). The C99 fragment is then cleaved by γ-secretase to form Aβ and APP intracellular domain (AICD) 5, 6. There can be multiple Aβ sizes, between 37-43 amino acids long, depending on where the γ-secretase cleaves. The two primary Aβ species are Aβ40 and Aβ42, with Aβ42 being more prevalent in plaques 5. These Aβ oligomers are highly toxic to neuronal culture *in vitro* 7, 8. Several mutations found in APP or presenilin (PSEN) proteins, dubbed familial Alzheimer's disease (FAD) mutations, lead to amyloidogenic processing of APP into Aβ 9, 10. Mouse models, such as the 5XFAD model, take advantage of these mutations to study AD in an *in vivo* model. The 5XFAD model has 3 APP and 2 PSEN1 mutations that cause rapid development of Aβ plaques leading to AD behavioral phenotypes, such as decreased learning and memory capabilities 11-13. This model has been extensively studied to look at the development and possible therapeutics for AD.

However, Aβ plaques are not thought to cause AD on their own. Multiple studies show that inflammation plays a large role in AD development. Recently, an amyloid-cascade inflammatory hypothesis has been brought forward to explain the underlying toxicity of Aβ 1, 14, 15. Inflammation in the brain is a double-edged sword. Activation of astrocytes and microglia can eliminate debris and pathogens 16; however, this activation can also have harmful consequences on the neuronal system 17. In neurodegenerative diseases, the equilibrium is disturbed, causing chronic inflammation and tissue atrophy 18. Inflammation is believed to contribute and exacerbate AD pathology and possibly even precede AD 1, 15, 19-22. Since inflammation plays such a large role in disease development, many therapeutics target inflammation rather than APP processing. One of these new potential therapeutics is immunomodulatory mesenchymal stem cells.

Recently, human mesenchymal stromal or stem cells (hMSCs) have drawn much attention in correcting neurological disorders due to their active migration, multipotent potentials, neuroprotective, immunomodulation and paracrine effect 23-25. The regenerative potentials of hMSCs have been observed in various neurodegenerative disease such as ischemic stroke 26, multiple sclerosis 27, traumatic brain injury 28, as well as AD 29, 30. However, hMSC based therapies exhibit a major disadvantage in that they lose their cellular homeostasis and stem cell functions during in vitro culture expansion, which is required for clinical applications 31, 32. To maintain cellular homeostasis and therapeutic quality, preconditioning strategies such as hypoxia and 3D aggregation culture of hMSCs have been developed 33, 34, but alternative therapeutic strategies with hMSCs are of great interest for AD treatment.

Though the mechanism of hMSC-mediated neurological recovery is not well understood, increasing body of evidence revealed that the therapeutic effects are mainly attributed to hMSC secretome and paracrine effects 35, 36. As part of hMSC secretome, extracellular vesicles (EVs) are a heterogeneous population of membrane-bound sacs that function in cell-to-cell communication. They have a diverse molecular payload that includes DNA, RNA, proteins, and lipids 37-39. EVs are secreted by all cells and are abundantly present in most bodily fluids 40. EVs are classified by size and subcellular origin 41. Apoptotic bodies are the largest, between 100-5000 nm in size, and are formed from apoptotic blebbing of the cell during cell death. Microvesicles are formed from plasma membrane shedding and are between 100-1000 nm in size. Exosomes, or small EVs (sEVs), are typically between 30-150 nm in size and are formed following budding and fusion events on the limiting membrane of the multivesicular body (MVB) to form intraluminal vesicles (ILVs). When the MVB fuses with the plasma membrane, the ILVs are then released into the extracellular space as exosomes 37, 38, 41, 42.

EVs can cross most barriers, including the blood-brain barrier (BBB), and have similar potency to their parental cells 43, 44. This ability makes MSC-derived EVs ideal potential therapeutics for diseases such as AD 30, 45-47. Recently, intranasal (IN) administration has been used for brain therapeutics since using intravenous (IV) administration causes a low amount of drugs or sEVs to get to the brain, as they go to other organs throughout the body 48, 49. Here we used bone marrow MSC-derived sEVs delivered via IN administration to test their effects on AD pathology and behavior. We used a two-month-old 5XFAD mouse model in this study and delivered the EVs every four days for up to four months. At four months of age there is high plaque load in these mice without treatment 50. We found that the hMSC-EV treatment significantly improved the 5XFAD mouse memory using various behavioral tests. Using immunohistochemistry (IHC), we also found that IN administration of sEVs significantly decreased the number of Aβ plaques in the hippocampus (HPC). Together, these data show promising results for developing MSC-derived EVs as a therapeutic for AD.

## Methods

### Cell culture

Standard frozen hBM-MSCs at passage 1 (P1) were obtained from the Tulane Center for Stem Cell Research and Regenerative Medicine. hBM-MSCs were isolated from the bone marrow of healthy donors ranging in age from 19 to 49 years based on plastic adherence, being negative for CD34, CD45, CD117 (all less than 2%) and positive for CD29, CD44, CD49c, CD90, CD105 and CD147 markers (all greater than 95%) and possessing tri-lineage differentiation upon induction *in vitro* 51, 52. hBM-MSCs were expanded with complete culture medium (CCM) composited with minimum essential medium-alpha (α-MEM, Cat#12000063, Thermo Fisher Scientific, Waltham, MA) supplemented with 1% Penicillin/Streptomycin (Cat#97063-708, VWR international, Radnor, PA) and 10% fetal bovine serum (FBS) (Cat#S11150, Atlanta Biologicals, Lawrenceville, GA) on 150-mm tissue culture petri dishes (Cat# 25382-442, Corning, Corning, NY) at a density of approximately 1,500 cells/cm^2^ in a standard 5% CO_2_ incubator at 37 ºC. The culture media were changed every three days. Cells were grown to 70-80% confluence and then harvested by incubation with 0.25% trypsin/EDTA (Cat# 25200056, Thermo Fisher Scientific, Waltham, MA) and re-plated for subculture up to passage 6 (P6). Cells from P5 and P6 were used in all experiments.

3D aggregation of hBM-MSCs were performed following previous publication (26). Briefly, hMSCs at 80-90% confluence were harvested and seeded in each well of ultra-low attachment (ULA) six-well plates (Cat# 29443-030, Corning, Corning, NY). Each well contains 1.0-2.0 × 10^5^ cells with 2 mL CCM with 10 % EV-depleted FBS. EV-depleted FBS was made by spinning FBS at 29000 rpm in a SW32 rotor for 20 h, then filtering the FBS in a 0.22 μm filter, being careful as to not disturb the pelleted EVs. Cells cultured on a tissue culture plate (TCP) were used as 2D control. The time interval for collecting conditioned medium is 48 h.

### Extracellular vesicle enrichment

The enrichment of EVs has previously been described 53, 54. Additionally, we have submitted all relevant data of our experiments to EV-TRACK database (EV-TRACK ID: EV210120) 55. Briefly, spent culture media underwent modified differential centrifugation, 500 x g for 5 min, 2000 x g for 10 min, 10000 x g for 30 min. Next, media was incubated with a 1:1 volume of 2X PEG solution (16% polyethylene glycol 6000, 1 M NaCl) overnight at 4 ºC. The next day, media was spun at 3200 x g for 60 min. Media was decanted, and the pellet was resuspended in sterile, filtered PBS, and a wash spin was performed at 100000 x g for 70 min. This pellet was then resuspended in sterile, filtered PBS, aliquoted and stored in -80 ^o^C for further downstream analysis or diluted for IN administration. The day of administration, EVs were thawed and diluted to the correct concentration. Once thawed, EVs were not re-frozen to prevent lysing. The EVs were used up to a month post-freezing.

### Electron microscopy

Electron microscopy was performed on the EV samples following ultracentrifugation. Grids were prepared as previously described 56.

### Immunoblot analysis

Whole-cell lysates were prepared by washing cells with PBS, pelleting, then lysing with radioimmunoprecipitation assay (RIPA) buffer as described previously 6. Equal protein of cell lysates and EV samples were measured by Pierce 660 nm Protein Assay (Invitrogen, 22,662) then loaded into an SDS 10% or 12% polyacrylamide gel. Western blot analysis was performed as described 57. Ponceau S stain (Sigma, P7170) was used to visualize total protein. Blots were probed with the following antibodies: TSG101 (4A10; Genetex), CD63 (TS63; Abcam), CD9 (MM2/57; Millipore), Syntenin-1 (S-31; Santa Cruz), Calnexin (H-70; Santa Cruz), Aβ (2454; Cell Signaling), GFAP (PA5-16291, ThermoFisher), rabbit anti-mouse IgG (Genetex, GTX213112-01), goat anti-rabbit IgG (Genetex, GTX213110-01). Blots were imaged using a Bio-Rad ChemiDoc imager and processed using ImageQuant TL v8.1.0.0 software and CorelDraw Graphic Suite X5.

### Nanoparticle tracking analysis

Nanoparticle tracking was performed using a Malvern NanoSight LM10 instrument, and videos were processed using NTA 3.4 software as previously described 58.

### Animal procedures

All animal procedures were carried out in accordance with the guidelines for the Florida State University Institutional Animal Care and Use Committee (ACUC), protocol number 1902, and all studies were performed in accordance with the recommendations in the National Institute of Health's Guide for the Care and Use of Laboratory Animals.

An equal number of 6-week-old male and female mice were obtained from Jackson Labs. This included 28 nontransgenic (NT) C57BL/6J and 28 transgenic (5XFAD) mice. After two weeks of acclimation to their housing and to handling, fourteen mice of each group received either IN sterile saline (5 µL) or IN EVs (20x10^8^ EVs in 5 µL) in saline in each of their nostrils every fourth day, as described in 59.

At four months of age, seven NT + seven 5XFAD saline controls and seven NT hMSC-EV + seven 5XFAD hMSC-EV treated mice were tested for memory performance using standard neurocognitive behavioral tests. One day following the conclusion of these tests, these 28 mice were terminally anesthetized, and both blood and brains were collected for circulating Aβ 1-42 levels and amyloid plaque detection. The remaining 28 mice continued IN treatments for two additional months. After this time, they underwent neurocognitive, behavioral assessments before sacrifice and tissue collections. Mice were checked daily and no toxic or adverse effects were observed in mice treated with hMSC-EVs.

All mice were euthanized by sterile intraperitoneal overdose injection of ketamine (Butler Schein; 100mg/kg) and xylazine (Vedco; 10mg/kg) and transcardially perfused first by 0.9% sterile saline, followed by 4% paraformaldehyde (PFA). Whole brains were removed and placed into 15ml of the same PFA fixative used for perfusion for 24 h at 4 °C. Brains were then placed into filtered 30% sucrose solution at 4 °C until the brains sank.

### Novel object recognition test (NORT)

This test was performed in a small, opaque, open field box (36cm long by 32cm wide by 29cm high), as described in 60. On the day before the experimental sessions, animals were habituated to the experimental room and NORT chamber for 10 min. Twenty-four h later, mice were given 10 min to interact in the testing chamber with two-identical objects. The animals were removed from the apparatus and given a 1-h training-to-testing interval. One of the now familiar objects was replaced with a novel object, which had been previously tested for comparable object manipulability and complexity interactions. The mice were again placed into the testing chamber and allowed to interact freely with the familiar and novel objects for 5 min. The familiar and novel objects were placed on opposite sides of the testing chamber for each trial, and the entire testing apparatus was thoroughly cleaned with 70% ethanol between each subject. The amount of time spent at both familiar and novel objects was determined via video analysis using a "within object area" scoring method. An animal was scored as interacting with the object when its nose was in contact with the object or directed at the object within ≤ 2 cm. Time spent standing, sitting, or leaning on the secured object was not scored as object interaction.

### Y-maze

Mice were placed in the center of the maze and allowed to explore for 7 min, with the number and sequence of arm choices being recorded. General activity was measured as the total number of arm entries, while basic mnemonic function was measured as a percent spontaneous alternation.

### Barnes Maze

Mice were placed in a circular platform, 150 cm above the ground, with 40 equidistantly spaced holes along its periphery, as described in 60. Under one hole was a box that the mice could use to escape the platform surface. The escape box location was moved between mice to a new randomly selected hole, but remained constant for a particular mouse across trials and days. Visual cues, located on curtains or the walls, could be used to learn and remember the location of the escape hole. Aversive stimuli (bright lights and wind from a small fan) were employed to motivate searching behavior. During a single 10-min maximum daily trial, the total number of errors (head pokes into non-escape holes) and latency to find the escape hole was recorded. Trials were conducted for four days.

### Analysis of behavioral tests

Videos were taken of the mice during behavioral testing and then randomly assigned to MV, AC, and SK, who were blinded to the group and genotype. Graphs and statistics were done in GraphPad. Y-maze and NORT tests were analyzed by a one-way ANOVA with Tukey's multiple comparison. Barnes maze was analyzed by a two-way ANOVA with Tukey's multiple comparison.

### MRI analysis of mouse brains

Perfused mice brains were placed into MR compatible vials and secured in a 1H birdcage coil before being placed in magnet. To determine the amyloid beta plaque deposition, 3D gradient-recalled echo fast low angle shot (GRE FLASH) scans, specifically gradient echo Fourier imaging tomography (GEFI-TOMO), were acquired on a 900-MHz, 21.1-T superconducting magnet located at the National High Magnetic Field Laboratory (NHMFL). 50-µm scans and 25-µm scans were acquired at 21.1-T to utilize higher spatial resolution to better visualize the plaque localization. Susceptibility-weighted MR images were utilized to assess plaque deposition, respectively, in preserved female 5xFAD and age-matched at 4 and 6 months.

### Dissection of mouse brain regions

The cerebellum and HPC was removed from three mice brains as described in 61. The rest of the brain was kept whole. HPC or the rest of the brain was lysed in RIPA lysis buffer with HALT protease inhibitor (78428; Thermo Fisher) using 500 μL per 10 mg of protein. Brains were homogenized thirty-five times then kept on ice for 30 min. Lysates were sonicated for 8 cycles (15 s on, 15 s off). Finally, lysates were centrifuged at 10000 x g for 20 min at 4^o^C. Supernatant was transferred to a new tube and kept at -80 ^o^C for later analysis.

### Cryotome sectioning

Frozen sections were cut coronally at a thickness of 40 µm using a Leica SM2010R sliding microtome. Slices were kept in PBS with 0.02% sodium azide until staining.

### Thioflavin S (ThioS) staining

ThioS stain was done as previously described 62. Briefly, slices were washed twice for 10 min in PBS. Next, slices were put in a blocking solution (TBS, 0.3% TritonX-100, 3% goat serum) for 2 h. Then slices were put in anti-NeuN (Millipore, ABN78) 1:1000 in TBS with 0.3% Triton X-100 (TBS-T) overnight. The next day, slices were washed three times in TBS-T, then put in anti-rabbit-TXRD (Southern Biotech, 4010-07) 1:300 in TBS-T overnight. The next day, 3 TBS-T washes were performed, then the slices were stained in 1% ThioS (Sigma, T1892) for 9 min. To de-stain, slices were washed twice with water, once with 70% ethanol, then twice with water. Slices were finally washed again in TBS then mounted onto Superfrost Plus slides (VWR, 48311-703). AF1+DAPI (EMS, 17970-125) mounting media was added, then the coverslip. Slides were imaged using a Zeiss Axio Imager.M2 the next day.

### 6E10 staining

Brain slices were mounted onto Superfrost Plus slides (VWR, 48311-703) (62, 63). Next, the slides were put in 4% PFA for 4 min, 2 TBS washes, then 70% formic acid (Sigma, F0507) for 2 min. Next, slides were washed twice with TBS, then put in TBS with 0.1% Triton-X for 15 min. After, slides were blocked in TBS-B (TBS, 0.1% Triton-X, 2% BSA) for 30 min. Anti-6E10, a Aβ 1-16 antibody, (Biolegend, 803015) at 1:1000 and anti-NeuN (Millipore, ABN78) 1:1000 were added in TBS-B for two days at room temperature. After washing in TBS, anti-mouse-alexa-488 (Southern Biotech, 1010-30) at 1:500 and anti-rabbit-TXRD (Southern Biotech, 4010-07) at 1:300 were added for 6 h, or up to overnight. After secondary, slides were washed in TBS, then AF1+DAPI (EMS, 17970-125) mounting media was added, then the coverslip. Slides were imaged using a Zeiss Axio Imager.M2 the next day.

### GFAP staining

Slices were washed twice for 10 min in PBS. Next, slices were put in a blocking solution (TBS, 0.3% Triton X-100, 3% goat serum) for 2 h. Then slices were put in anti-GFAP (Invitrogen, PA5-16291) 1:1000 in TBS-T for two days. Next, slices were washed three times in TBS-T, then put in anti-rabbit-594 (Abcam, ab96985) 1:500 in TBS-T overnight. The next day, 3 TBS washes were performed, then the slices were stained in 1% ThioS (Sigma, T1892) for 9 min. To de-stain, slices were washed twice with water, once with 70% ethanol, then twice with water. Slices were finally washed again in TBS then mounted onto Superfrost Plus slides (VWR, 48311-703). AF1+DAPI (EMS, 17970-125) mounting media was added, then the coverslip. Slides were imaged using a Zeiss Axio Imager.M2 the next day.

### Analysis of IHC images

Images were deidentified with numbers and analyzed by blinded co-authors SK and SC. Twenty images were taken for ThioS and 6E10 quantification throughout the HPC. Twenty-five images were taken throughout the brain for GFAP and ThioS colocalization. Data were graphed and analyzed using GraphPad. Statistics were determined using Welch's t-test.

### Staining of EVs with a lipophilic dye

After PEG precipitation of EVs, the EVs were stained with a Vybrant DiO stain (Thermo Fisher, V22886) at 1:500 concentration for 30 min at 37 ℃. Next, EVs were washed with a 100000 x g ultracentrifugation wash for 70 min, leaving any unbound dye to be discarded. Mice were given IN administration of EVs or saline control with DiO stain subjected to the same DiO staining and centrifugation protocol as EVs. After 24 h, mice were sacrificed, and tissue was collected. After sectioning 10 μM sections, slices were imaged on the Keyence BZ-X710 fluorescence microscope.

## Results

### Characterization of MSC-derived EVs

**Figure 1:**
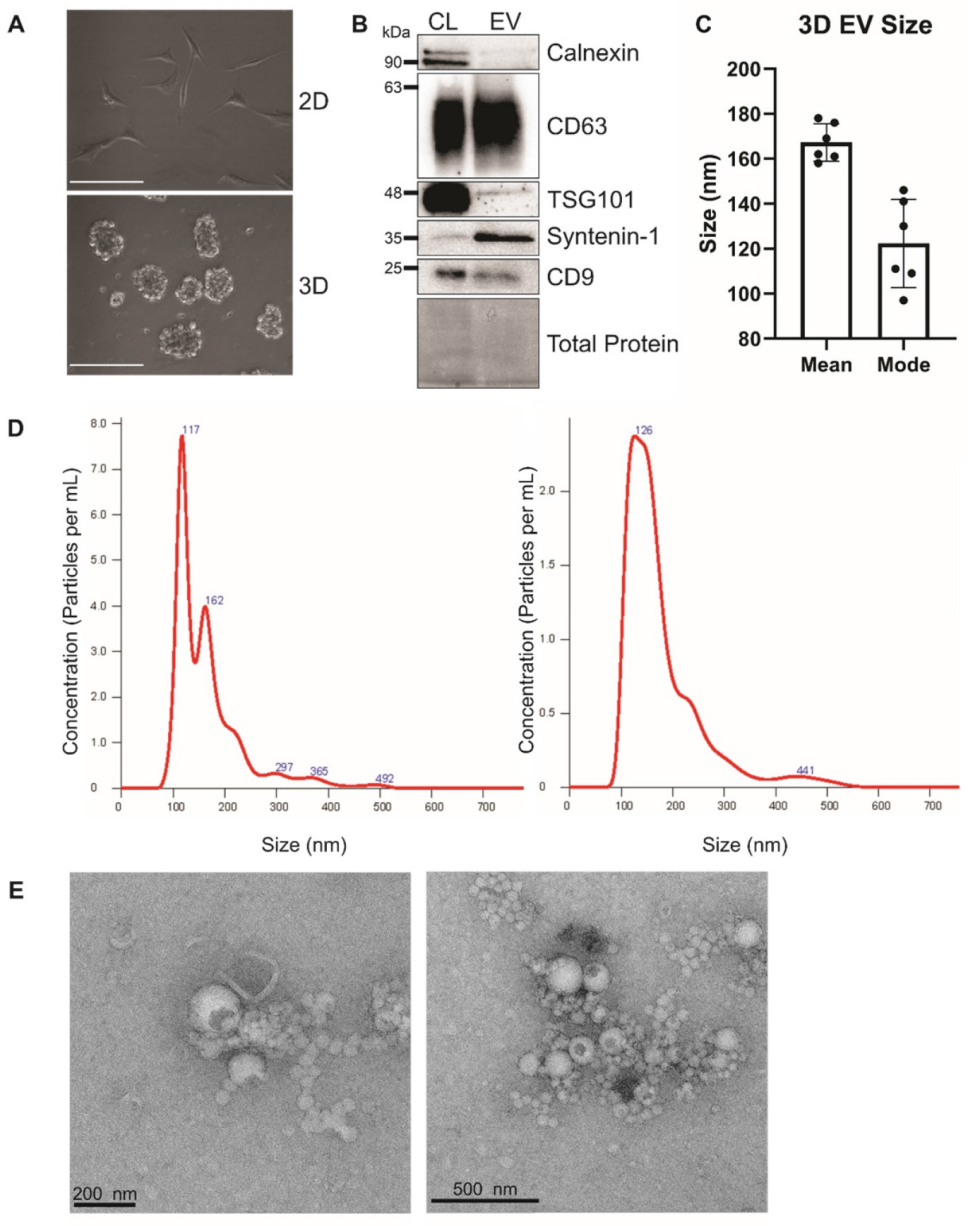
Characterization of 3D hMSC small EVs. (A) Image of 2D versus 3D cell growth. Scale bar = 100 μm. (B) Immunoblot analysis of hBM MSC-derived sEVs. **(C and D)** Nanoparticle tracking analysis of hMSC-EVs. (E) Electron microscopy of sEVs.

Human bone marrow-derived MSCs were grown in ultra-low attachment surface plates to form three dimensional human mesenchymal stem cell (3DhMSC) aggregates (**Figure 1A**). Small EVs (sEVs) were harvested from the media using the ExtraPEG method 51. The next day, media was spun at 3200 x g for 1 h. sEVs were characterized according to MISEV 2018 recommendations 64. sEVs were depleted in ER-enriched protein Calnexin and enriched in tetraspanin CD63 and ESCRT accessory protein Syntenin-1 (**Figure 1B**). Interestingly, tetraspanin protein CD9 was not increased in these sEVs. Nanoparticle tracking analysis (NTA) and transmission electron microscopy (TEM) were performed to determine the size and morphology of the 3D sEVs (**Figure 1C-D**).

To determine the migratory ability of sEVs in BBB via IN administration, sEVs were stained with a Vybrant DiO lipophilic dye, and 2x10^9^ sEVs in 5 µL or 5 µL of saline with Vybrant dye were administered IN to 4-month-old mice. Twenty-four ho later, mice were euthanized, and brains were harvested. After sucrose cryo-protection, 10 µm slices were taken through the pre-frontal cortex (PFC) and imaged using a Keyence fluorescent microscope. The saline-treated mice had little or no fluorescent signal, while the EV-treated mice had a high signal (**Supp. Figure 1**). This observation demonstrated the feasibility of hMSC-EV delivery through IN administration.

### MSC-derived EVs ameliorate AD behavioral phenotypes in 5XFAD mice

For evaluation of cognition and markers of pathology after IN sEVs treatment, two-month-old 5XFAD mice were administrated with 2x10^9^ 3D MSC- EVs in 5 µL of PBS or 5 µL saline. After two months of regular treatment, the first group of mice underwent a battery of behavioral tests to assess spatial and non-spatial learning and memory. 5XFAD mice have been previously shown to start developing plaques at four months of age 11. However, plaque density is low and cognitive deficits typically are not severe until six months of age (**Supplemental Figure 2**) 48. Thus, we first assessed six-month old 5XFAD mice that had been continuously treated for four months with 3D-hMSC-EVs.

**Figure 2:**
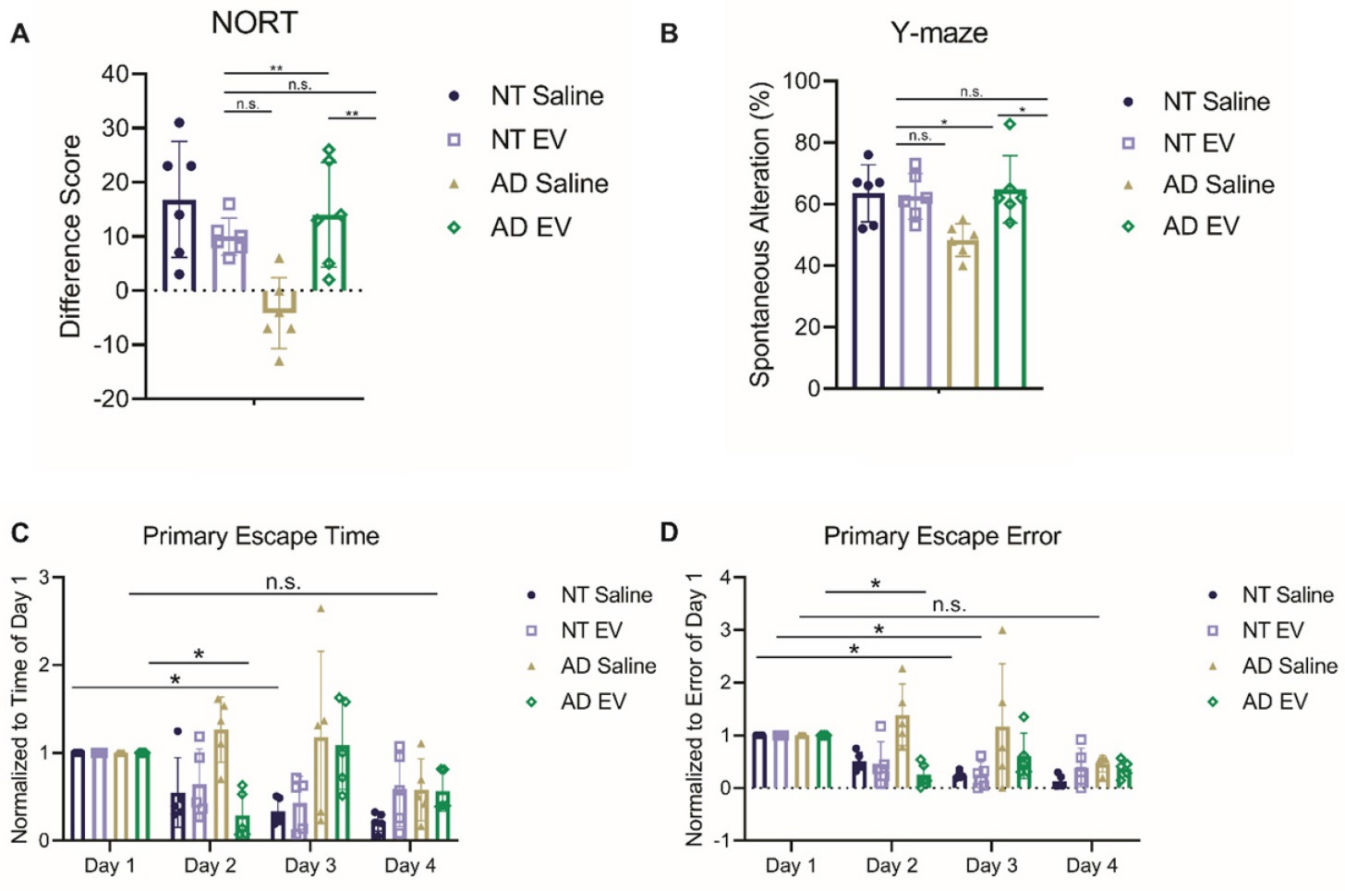
Treatment with hMSC-derived EVs ameliorate cognitive decline of 5XFAD mice. **(A)** Mice were tested for memory using novel object recognition test (NORT). hMSC-EV treated 5XFAD mice performed significantly better than saline treated 5XFAD mice. N=6.** (B)** Y-maze test was used to determine spatial working memory. hMSC-EV treated 5XFAD mice performed significantly better than saline treated 5XFAD mice. N=6. Finally, mice were tested for spatial learning and memory using the Barnes maze. hMSC-EV treated 5XFAD mice performed significantly better on both **(C)** primary escape time and **(D)** primary escape error. One-way ANOVA was used to determine the statistical significance of A and B. Two-way ANOVA was used to assess the significance of C and D. N=5. *P < 0.05; **P < 0.01.

The first test administered was the novel object recognition test (NORT) for assessing object memory. Mice with intact learning and memory will interact with the novel object more than the familiar one 65. Interaction time varied significantly across groups (**Figure 2A**; F_(3,20)_ = 7.923, *p* = 0.0011). Specifically, the saline-treated 5XFAD mice interacted more with the familiar object, performing significantly worse than EV-treated 5XFAD mice and NT mice (ps < 0.01), but did not differ from hMSC-EV treated NT mice (p = 0.4792).

Next, the spontaneous alteration was assessed using a Y-maze. Spatial alternation performance is thought to reflect spatial working memory 66. Spatial alternation performance varied significantly across groups (**Figure 2B**; F_(3,20)_ = 4.901, *p* = 0.0103). Specifically, the saline-treated 5XFAD mice performed significantly worse than all other groups (ps < 0.05), while the hMSC-EV treated 5XFAD mice did not differ from NT mice (p = 0.9928).

Finally, the Barnes maze test was used to analyze spatial learning and memory. Over the 4-day trial, mice with intact spatial learning and memory will find the escape hole in less time (primary escape time) and look in fewer spots that are not the escape hole (primary escape error) 67. Escape time significantly decreased across training (**Figure 2C-D**; Fs_(3,64)_ = 5.86, ps = 0.0013), varied as a function of group (Fs_(3,64)_ = 6.44, ps = 0.0007), and there was a significant interaction between day and genotype (Fs_(9,64)_ = 2.774, ps = 0.0083). Similarly, errors varied significantly across day (Fs_(3,64)_ = 7.911, ps = 0.0001), and group (Fs_(3,64)_ = 9.824, ps < 0.0001), and there was a significant interaction between day and genotype (Fs_(9,64)_ = 2.414, ps = 0.0199) both varied significantly across day. Post-testing, corrected for repeated measures, showed that the NT mice and EV-treated 5XFAD mice had a significant decrease in escape time and error by the second or third day (NT Saline Day 1 vs. Day 3 p < 0.01; NT hMSC-EV Day 1 vs. Day 3 p < 0.05; 5XFAD hMSC-EV Day 1 vs. Day 2p < 0.05). However, saline-treated 5XFAD mice did not have a significant reduction in escape time or error between for any day (p-value = 0.17).

### Treatment with 3D-MSC-EVs decreases amyloid plaque deposition

To determine how treatment with sEVs may ameliorate cognitive decline in these mice, we next looked at levels of amyloid beta in the brain. Each group of mice were euthanized, and brains were harvested to perform immunohistochemistry (IHC) to determine the effect of MSC EVs on AD pathology. Brain slices were stained with Thioflavin S (ThioS) or 6E10 (Aβ 1-16 specific antibody).

**Figure 3:**
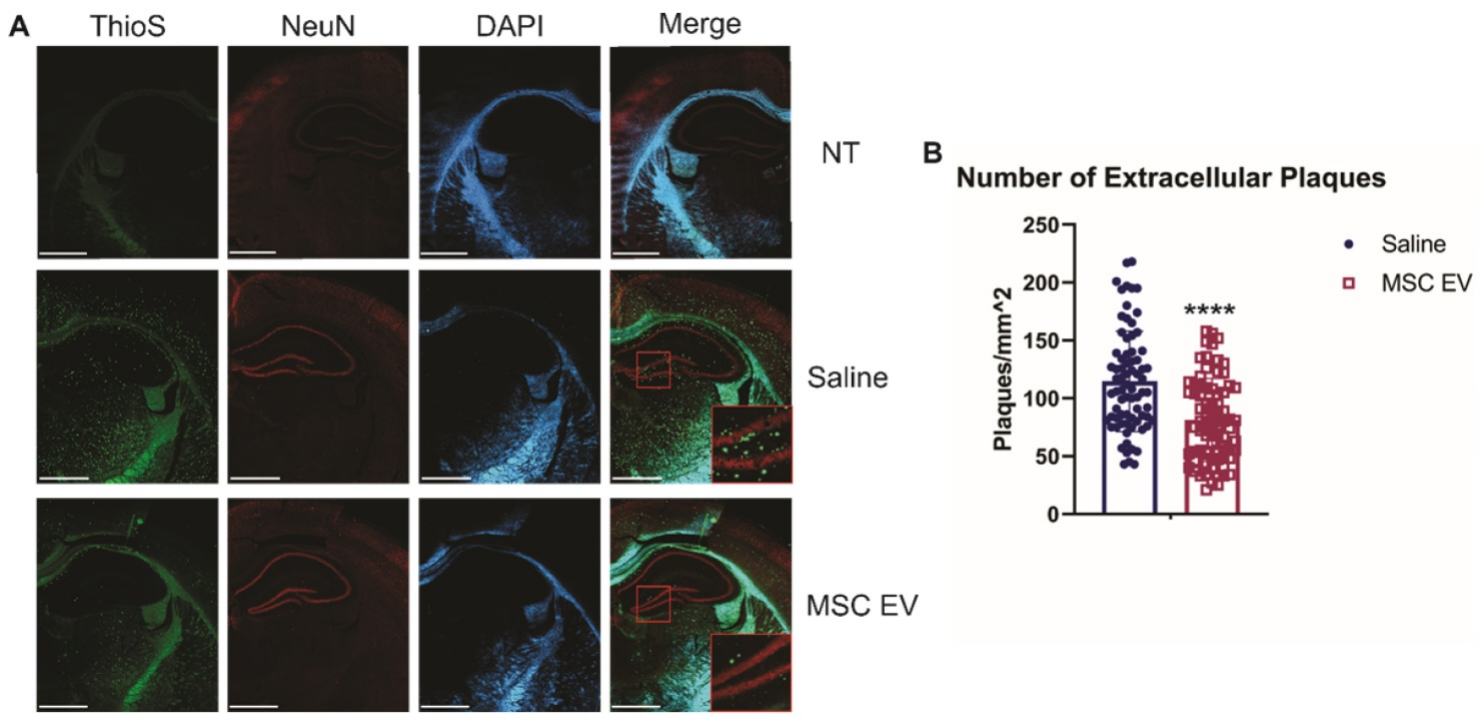
Intranasal administration of hMSC-EVs significantly decreases amyloid plaques in the HPC of 5XFAD mice. (A) Representative staining of brains with thioflavin S (ThioS), NeuN, and DAPI. Scale bar = 1 mm. (B) 20 one mm^2^ images were taken from the HPC of 4 different mice and plaque density was quantified using ImageJ. ****P < 0.0001.

Though intracellular levels of Aβ are associated with AD, it is the development of extracellular plaques that is one of the hallmarks of AD and more readily compared between rodents and humans. Thus, we first assessed the density of extracellular plaques using the ThioS stain. hMSC-EV treated 5XFAD mice had significantly less plaque deposition in the HPC compared to the saline-treated group (p-value < 0.0001; **Figure 3**). Next, we used the 6E10 antibody to assess the density of cells positive for intracellular Aβ and found no significant difference in intracellular levels in the HPC between the saline and hMSC-EV treated 5XFAD mice (p-value = 0.104**; Supplemental Figure 2**). The lack of effect on HPC positive cell density despite improved cognition is not entirely surprising, since intracellular levels of Aβ can remain constant across age points in mouse models of AD both before and after the emergence of cognitive deficits 68. However, we did find a significant decrease in Aβ positive cell density in the rest of the cortex (p < 0.0001; **Supplemental Figure 3**).

**Figure 4:**
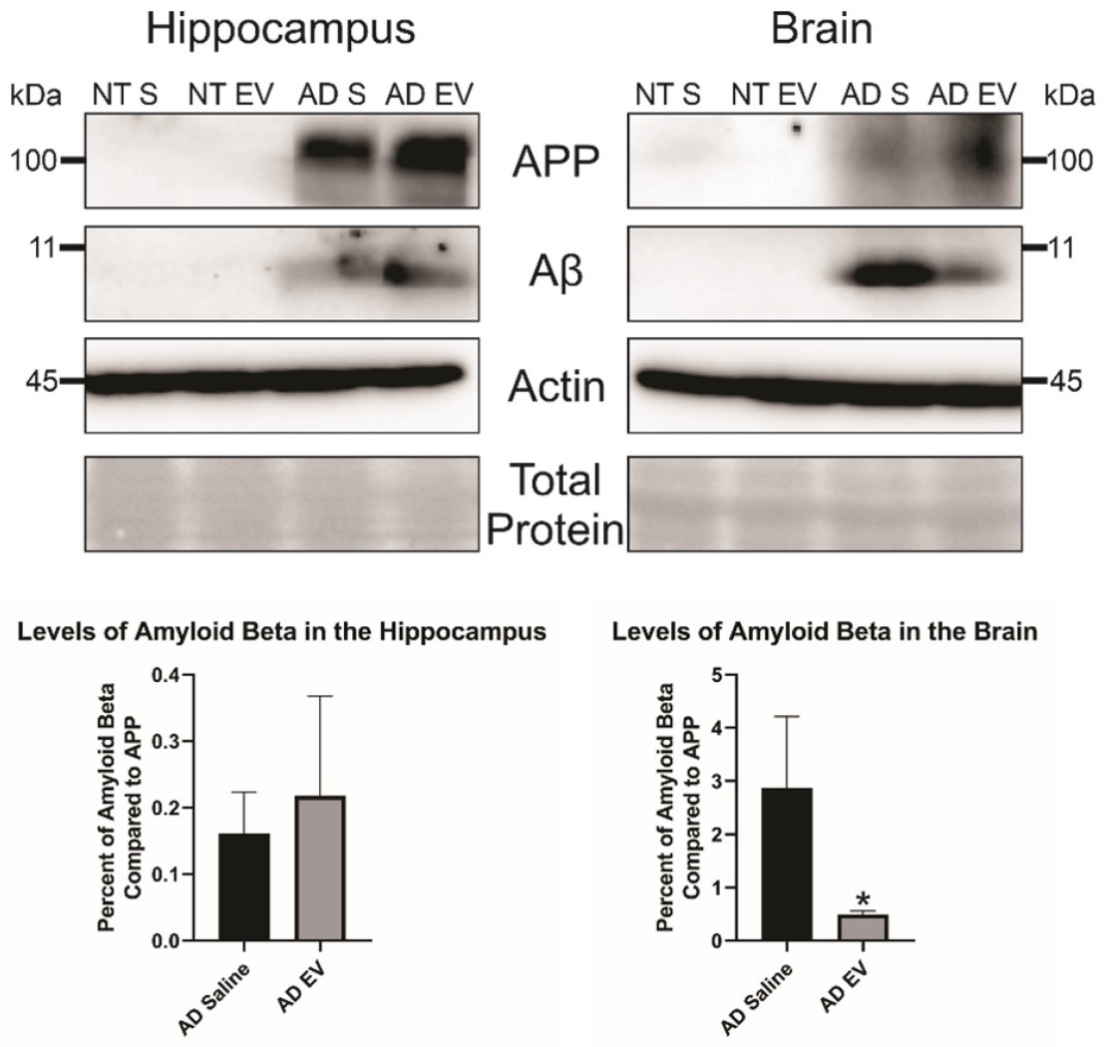
Levels of amyloid beta are significantly decreased in the brain of 5XFAD mice after treatment with hMSC-EVs. Three mice brains had the HPC dissected out, then HPC lysate or the rest of the brain lysate was run and analyzed by immunoblot assay. *P < 0.05.

Additionally, we wanted to look at total Aβ levels in both the hippocampus and the brain. We took three mice brains and dissected the hippocampus from the rest of the brain and ran a western blot with equal protein to determine Aβ levels in the brain. We found no difference in Aβ levels in the HPC between the saline and hMSC-EV treated mice (p-value = 0.298). However, there was a significant decrease of Aβ in the remainder of the whole brain (the brain material that remained after removing the HPC) of the hMSC-EV-treated mice (p-value = 0.0453; **Figure 4**).

### 3D hMSC EV treatment alters GFAP inflammatory marker levels in 5XAD mice

**Figure 5:**
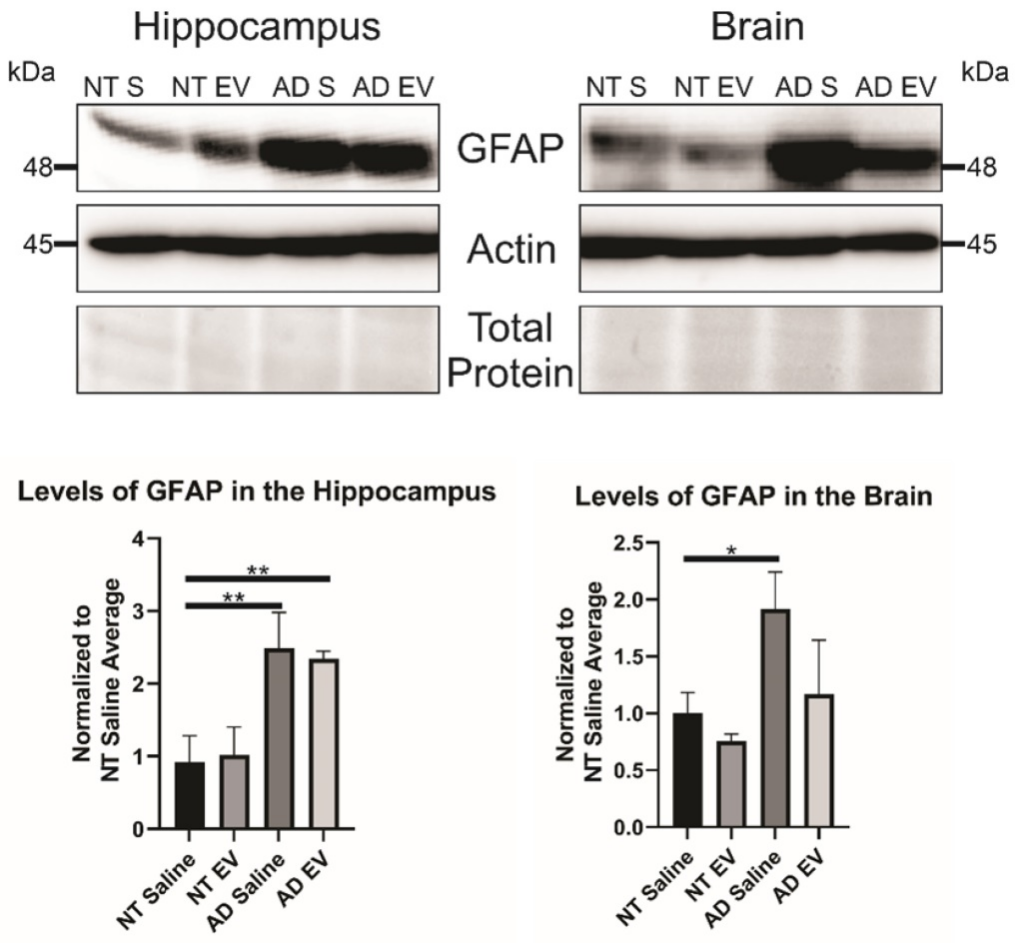
Treatment of 5XFAD mice with hMSC-EVs decreases the amount of GFAP in the brain. Three mice brains had the HPC dissected out, then HPC lysate or the rest of the brain lysate was run and analyzed by immunoblot assay. *P < 0.05; **P < 0.01.

**Figure 6:**
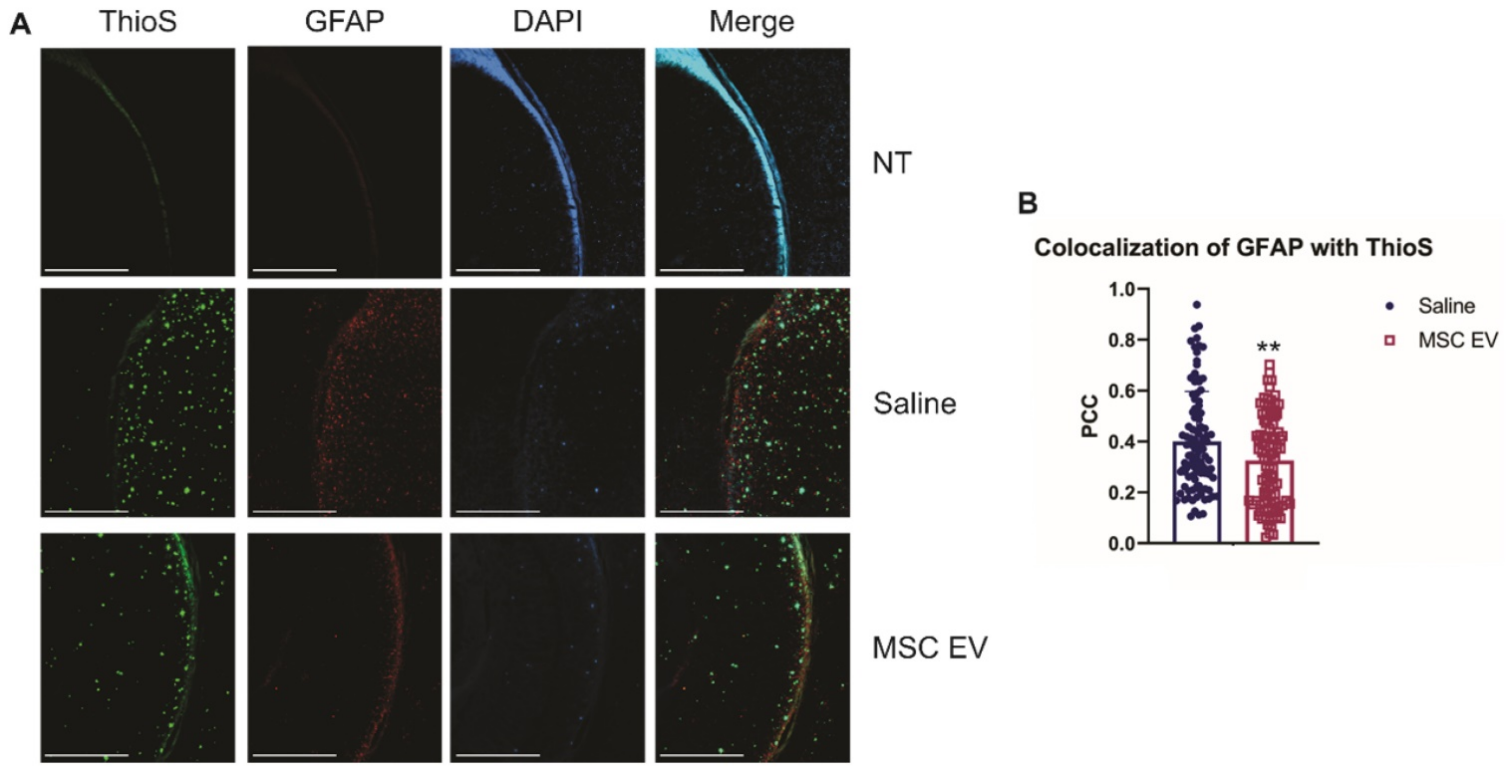
hMSC-EV treatment decreases colocalization between reactive astrocytes and amyloid plaques.** (A)** Representative immunohistochemistry images of brain slices stained with ThioS, GFAP, and DAPI. Scale bar = 1 mm. **(B)** We took 25 one mm^2^ images throughout the brain, and Pearson's correlation coefficient (PCC) was obtained through ImageJ analysis to determine colocalization of GFAP and ThioS. **P < 0.01.

As stated previously, recent research has indicated that inflammation, rather than Aβ alone, plays an important role in AD development. Therefore, we hypothesized that the hMSC-EV treatment may be inhibiting inflammation in the brain associated with AD pathogenesis. Glial fibrillary acidic protein (GFAP) is an intermediate filament protein that is a marker for astrocytes. It is known to be activated upon brain damage or degradation 69. AD pathology has been shown to increase astrogliosis and expression of GFAP 70, 71. Reactive astrocytes have been found to interact with amyloid plaques inside the brain closely, and there is a correlation between GFAP levels and the density of plaques 72, 73. Consistent with previous reports, we found that there was an increase in GFAP levels in saline-treated 5XFAD mice compared to NT saline treated mice (NT Saline vs. 5XFAD Saline p-value = 0.0033 in the HPC, NT Saline vs. 5XFAD Saline p-value = 0.0247 in the brain; **Figure 5**) 74. GFAP levels were also higher in EV-treated 5XFAD mice than NT hMSC-EV treated mice for the HPC (p-value = 0.009), but not the remainder of the brain (p-value = 0.396). EV-treated 5XFAD mice did not have significantly less HPC GFAP than saline-treated 5XFAD mice (p-value = 0.9592) but did have a slight though non-significant decrease in the rest of the brain (p-value = 0.064). Additionally, EV-treated mice had reduced GFAP levels that were not different from the NT controls (p-value = 0.9012). Finally, we stained brains with ThioS and GFAP to determine the level of interaction between astrocytes and amyloid plaques. Previous research has shown that in late-stage AD, there is a high level of activated astrocytes that surround plaques, which leads to high levels of inflammation 69, 70, 72. As expected and similar to what was observed in Figure 5, low levels of GFAP were detected in the brains of NT mice which should have low levels of astrocyte activation and inflammation. We found a significant decrease in colocalization between GFAP and ThioS when mice were treated with MSC EVs (p-value = 0.0047; **Figure 6**).

## Discussion and Conclusion

Presently, there is no cure for AD. MSC and MSC-derived EVs have shown to be promising therapeutics for a multitude of diseases. However, when given intravenously (IV), MSC EVs have been shown to accumulate at the liver and spleen instead of the brain 75. Previous studies have altered MSC EVs to increase their affinity towards the brain; however, it is unknown whether these alterations can have any adverse effects in humans 76. In this current study, we employed IN administration of hMSC EVs and found that EVs can cross the BBB and enter the brain without further altering EV properties, as others observed 77. We also found that treatment with hMSC EVs ameliorate cognitive decline in a 5XFAD mouse model. Additionally, we discovered that there was a decrease in the number of extracellular plaques in transgenic mice after hMSC-EV treatment. Finally, we found that IN treatment with hMSC-EVs changes localization of inflammatory marker GFAP.

In our study, we used EVs from human bone marrow MSCs grown in 3D aggregation culture. Although PEG precipitation of EVs has been shown to have co-purifying non-EV material, the ultracentrifuge wash step has been found to clear some of these contaminants 78. PEG precipitated MSC-sEVs has already been used in clinical settings 79, 80. Interestingly, the EVs were enriched in Syntenin-1 and CD63, but not CD9 and TSG101. Recent studies have found that CD9 might be more enriched in small microvesicles due to its localization on the plasma membrane, whereas CD63 is found mostly in exosomes since it is highly enriched in MVBs 81. Recent studies have also found that exosome formation involves the syndecan-alix-syntenin-1 pathway 82. Thus, our results indicated that the 3D aggregation culture may increase the production of exosomes or small EVs produced in an ESCRT-independent manner compared to other EVs. Although the EV biogenesis in 3D aggregation of hMSCs is not the focus in this study, future research will be needed to elucidate the biological nature of these sEVs.

In this study, we employed a 5XFAD mouse model that mimics amyloid-beta (Aβ) plaque deposition seen in humans with AD. However, phosphorylated tau tangles have also been shown to be linked to AD 83. Hyperphosphorylated tau protein similarly aggregates like Aβ in an almost prion-like manner 84. These tau-tangles cannot be degraded or cleared and have also been found to be in plaques in the brain and lead to neuron and tissue death 85. Additionally, they can also lead to hyper inflammation in the brain 86. Here, we chose to take a mechanistic approach and focus on an Aβ aggregation model, which also provide certain inflammatory processes seen in humans 87. However, future studies should assess the effects of this potential treatment on animal models with Tau aggregation.

We found a decrease in cognitive decline in learning and memory in the 5XFAD mice treated with MSC-derived EVs compared to saline in this study. Similarly, we observed a reduction in amyloid plaques in the HPC of the EV-treated mice, along with a decrease of Aβ in the rest of the brain. While it is tempting to speculate that plaque reduction may be responsible for the intact learning and memory we observed, the exact relationship between intact learning and Aβ accumulation is not known. Aβ plaques are thought to have a multitude of causes. Initially, plaques were believed only to be caused by amyloidogenic processing of APP in neurons and that plaques then activate an inflammatory response that drives microglia and astrocytes to clear Aβ from the brain 88-90. However, recent research has found that glial cells can also produce Aβ and that this Aβ can also lead to plaque development 90, 91.

In addition to assessing Aβ plaque accumulation, we also monitored the levels of GFAP, which is a marker for activated astrocytes. We found a decrease in colocalization between GFAP and amyloid plaques in the hMSC-EV treated mice, along with a reduction in total GFAP levels in the brain. GFAP is a marker that is elevated in both mouse and human brains during AD development 92. Activated astrocytes secrete cytokines to recruit microglia and other glial cells to the site of plaques to aid in Aβ clearance. However, chronic secretion and activation by cytokines are hypothesized to increase the production of Aβ by astrocytes 93. Additionally, chronic inflammation and secretion of cytokines induce cell death of both neurons and glial cells 94. This may be caused by reactive astrocytes producing reactive oxygen species (ROS) 95, 96. Our results imply that the hMSC-EV treatment may reduce chronic inflammation in 5XFAD mice and possibly help facilitate the Aβ clearance as well as maintain proper tissue microenvironment for neurological restoration.

Modulation of inflammation in AD development has been a recent source of research to develop potential therapeutics. Recent studies have aimed to elucidate the line at which glial cells become harmful in AD progression. We believe that hMSC-derived EVs exhibited similar immunomodulatory capabilities as hMSCs and are tilting the inflammation process in AD towards a favorable outcome, leading to a decrease in plaque load and less cell death. This process slows down AD progression, causing a reduction in cognitive decline. Indeed, further research is needed to elucidate the exact mechanism of these hMSC-EVs therapeutic capabilities and how they can aid in both slowing down AD and possibly aid in other chronic inflammatory diseases.

## Supplementary Material

Supplementary figures.

## Abbreviations

AD: Alzheimer's disease
Aβ: amyloid beta
APP: amyloid precursor protein
AICD: APP intracellular domain
ESCRT: endosomal sorting complex required for transport
EV: extracellular vesicle
FAD: familial Alzheimer's disease
GFAP: glial fibrillary acidic protein
HPC: hippocampus
ILV: intraluminal vesicle
IN: intranasal
MSC: Mesenchymal stem cell
MVB: multivesicular body
NT: non-transgenic
NORT: Novel object recognition test
PBST: PBS with 0.3% Triton-X
PEG: polyethylene glycol
PFC: pre-frontal cortex
PSEN: presenilin
sEV: Small EV
sAPPβ: soluble APP β
TBST: TBS with 0.3% Triton-X
ThioS: Thioflavin S
