# Supplementary material for: Mesenchymal stem cell-derived extracellular vesicles ameliorate Alzheimer's disease-like phenotypes in a preclinical mouse model
Source: Theranostics. 2021 Jul 13;11(17):8129–42. doi: 10.7150/thno.62069 (PMC8344012; doi:10.7150/thno.62069)
Supplement: Supplementary file 1 — Supplementary figures. [file thnov11p8129s1.pdf]

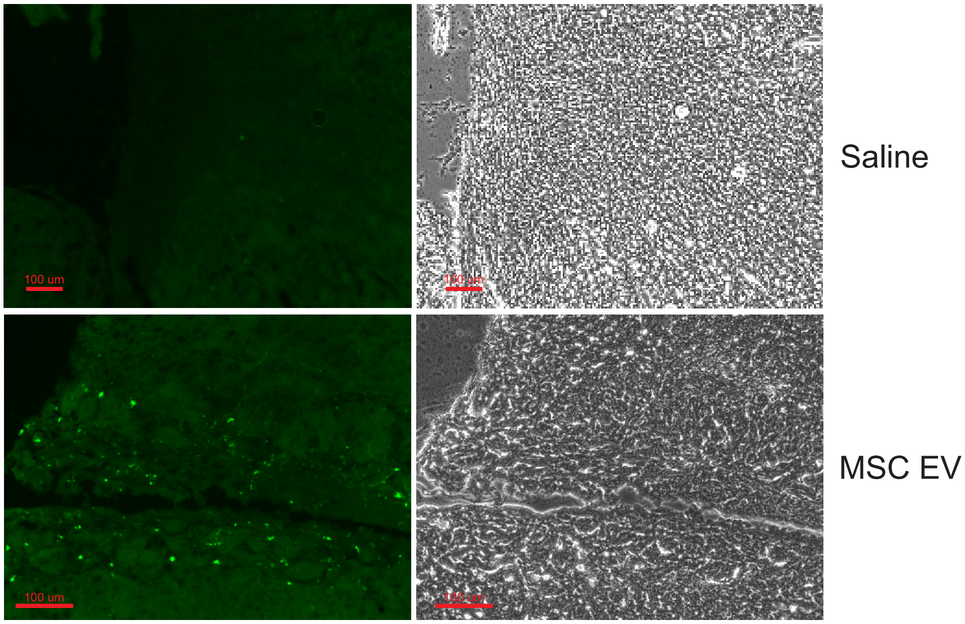


Supplemental Figure 1: Intranasal treatment of hMSC-derived EVs cross the blood-brain barrier. Two-month-old mice were given saline with Vybrant Dio dye, or EVs stained with Vybrant Dio. 24 h after administration, mice were euthanized, and sections were taken and imaged with Keyence BZ-X800. Right side imaged with fluorescence; left side imaged with brightfield.

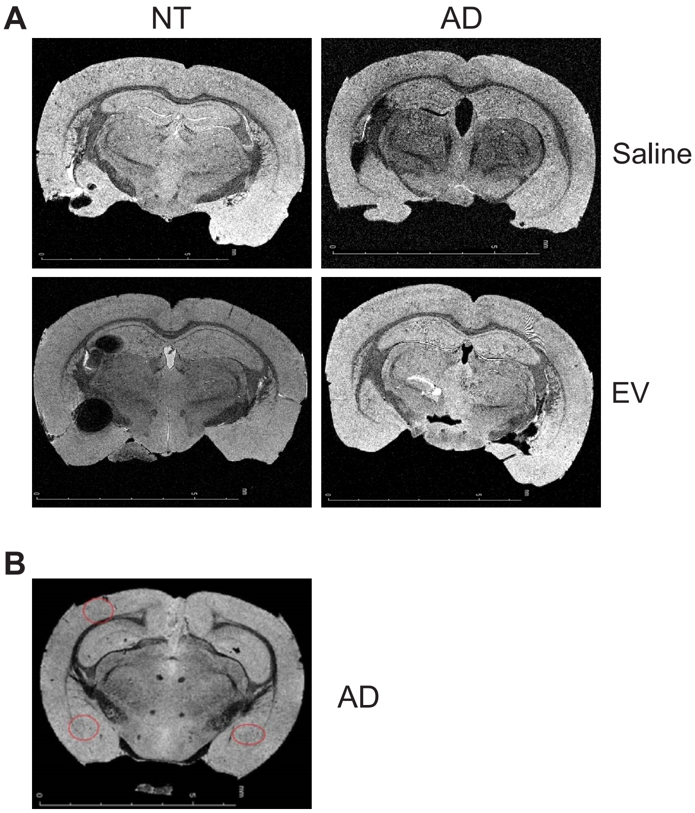


Supplemental Figure 2: MRI shows a lack of plaque formation at four months of age. (A) MRI images of four-month-old NT or 5XFAD mice with saline or hMSC-EV treatment. (B) MRI image of a 6-month-old 5XFAD mouse. Plaques are circled in red.

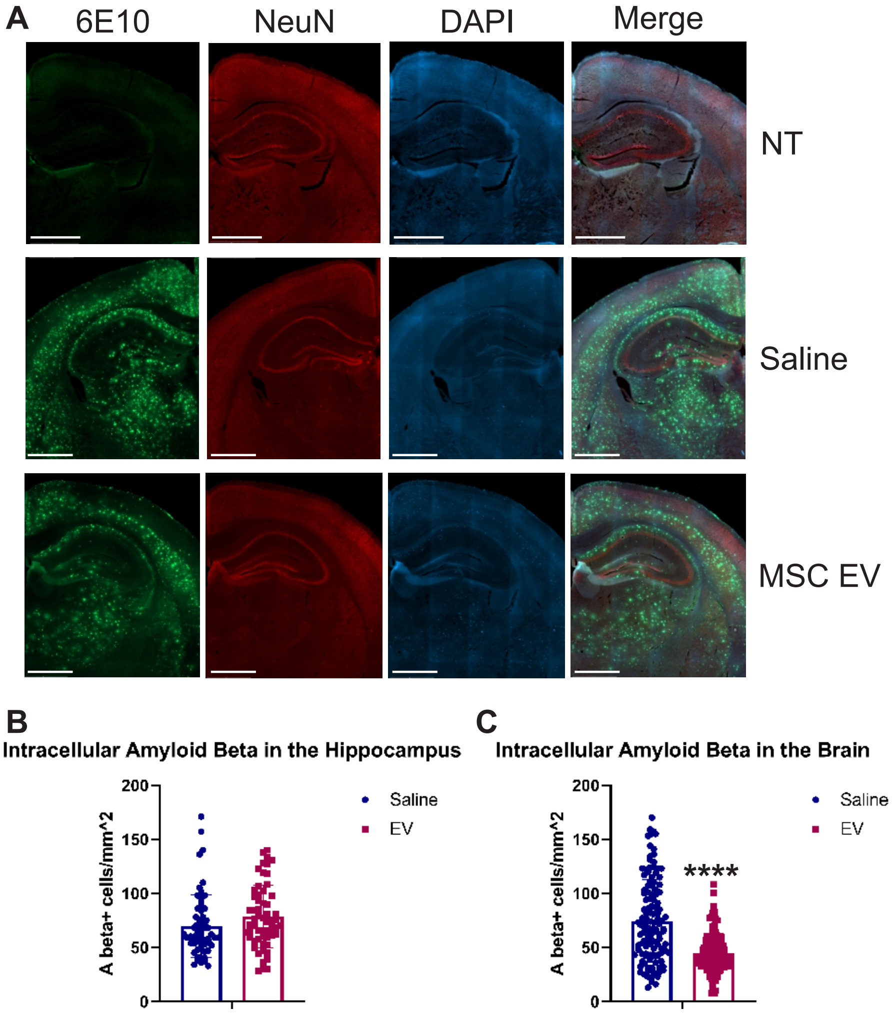


Supplemental Figure 3: Treatment with hMSC-EVs decreased intracellular amyloid-beta outside of the HPC but not in the HPC of 5XFAD mice. (A) Representative staining of brains with 6E10, NeuN, and DAPI. Scale bar = 1 mm. (B) 20 one mm2 images were taken from the HPC of 4 different mice and quantified using ImageJ. (C) 30 one mm2 images were taken throughout the cortex, excluding HPC, and quantified using ImageJ. ****P < 0.0001.

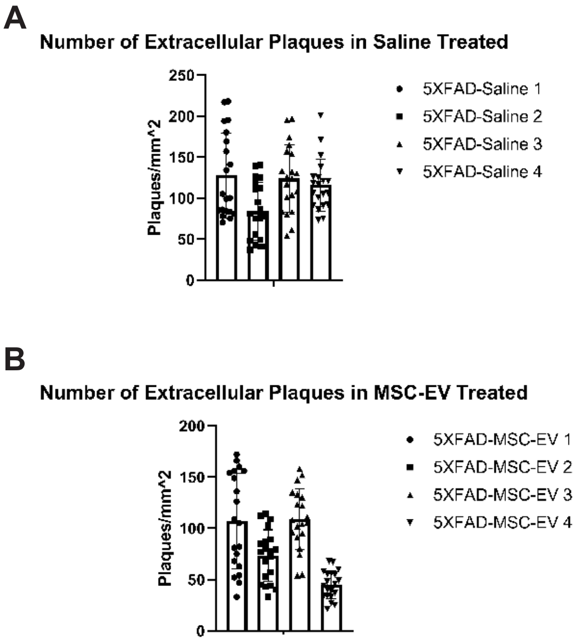


Supplemental Figure 4: The number of extracellular plaques for each mouse. 20 one mm2 images were taken from the HPC of 4 different mice and plaque density was quantified using ImageJ. (A) Number of plaques per mouse in the saline treated. (B) Number of plaques per mouse in the hMSC-EV treated.
